# In Situ RheoNMR Correlation of Polymer Segmental Mobility with Mechanical Properties during Hydrogel Synthesis

**DOI:** 10.1002/advs.202104231

**Published:** 2021-12-11

**Authors:** Christian Fengler, Jonas Keller, Karl‐Friedrich Ratzsch, Manfred Wilhelm

**Affiliations:** ^1^ Institute for Chemical Technology and Polymer Chemistry Karlsruhe Institute of Technology (KIT) Karlsruhe 76131 Germany; ^2^ Bruker BioSpin GmbH Ettlingen 76275 Germany

**Keywords:** crosslinks, hydrogels, molecular dynamics, networks, NMR relaxometry, radical polymerizations, rheology

## Abstract

Understanding polymer gelation over multiple length‐scales is crucial to develop advanced materials. An experimental setup is developed that combines rheological measurements with simultaneous time‐domain ^1^H NMR relaxometry (TD‐NMR) techniques, which are used to study molecular motion (<10 nm) in soft matter. This so‐called low‐field RheoNMR setup is used to study the impact of varying degrees of crosslinking (*DC*) on the gelation kinetics of acrylic acid (AAc) and *N*,*N*′‐methylene bisacrylamide (MBA) free radical crosslinking copolymerization. A stretched exponential function describes the *T*_2_ relaxation curves throughout the gelation process. The stretching exponent *β* decreases from 0.90 to 0.67 as a function of increasing *DC*, suggesting an increase in network heterogeneity with a broad *T*_2_ distribution at higher *DC*. The inverse correlation of the elastic modulus *G*′ with *T*_2_ relaxation times reveals a pronounced molecular rigidity for higher *DC* at early gelation times, indicating the formation of inelastic, rigid domains such as crosslinking clusters. The authors further correlate *G*′ with the polymer concentration during gelation using a *T*_1_ filter for solvent suppression. A characteristic scaling exponent of 2.3 is found, which is in agreement with theoretical predictions of *G*′ based on the confining tube model in semi‐dilute entangled polymer solutions.

## Graphical abstract

The crosslinking copolymerization of acrylic acid (AAc) and *N*,*N*′‐methylene bisacrylamide (MBA) is studied using a novel experimental setup that combines rheology with time‐domain NMR relaxometry. The in situ correlation of the elastic modulus with transverse relaxation times reveals a distinct increase of network heterogeneity at higher crosslinker concentrations.

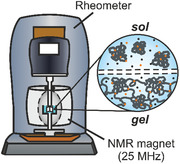

## 1 Introduction

Polymer gels based on acrylic acid (AAc) are viscoelastic materials that are prominent for their high absorption capacities. They are often referred to as superabsorbent polymers (SAPs) and are most commonly used in disposable hygiene products such as diapers.^[^ ^1^ ^]^ SAPs are further used in ion exchange resins, water treatment, controlled drug‐delivery systems, and as flow modifying additives in concrete.^[^ ^2^, ^3^, ^4^, ^5^ ^]^ Poly(acrylic acid) (PAAc) based SAPs are typically synthesized by free radical crosslinking copolymerization of AAc using *N*,*N*′‐methylene bisacrylamide (MBA) as a bifunctional crosslinker.^[^ ^1^ ^]^ The reaction mechanism and the impact of the reaction conditions on the network structure have been extensively studied.^[^ ^6^, ^7^, ^8^, ^9^ ^]^ These studies conclude that the network structure has a structural complexity on multiple length‐scales, ranging from connectivity defects (<10 nm), such as dangling ends and loops, inhomogeneous spatial distributions of crosslinks (10–100 nm) to microscopic density fluctuations.^[^ ^10^, ^11^, ^12^, ^13^, ^14^ ^]^ Various application‐relevant mechanical properties such as fracture resistance, mechanical strength, and permeability are affected by a complex interplay of those structural elements, and therefore, a deeper understanding of their time‐evolution during gelation is desirable.

Rheological measurements, which determine the viscoelastic response to a defined deformation, have been used to study the impact of synthetic parameters on the macroscopic mechanical properties. For instance, the time‐evolution of the elastic modulus during gelation was measured for varying crosslinker concentrations. It has been found that the crosslinking efficiency decreased at higher crosslinker concentrations.^[^ ^15^, ^16^, ^17^, ^18^ ^]^ This decrease in crosslinking efficiency was attributed to the formation of network defects on the nanoscopic level, such as the formation of nanogels and connectivity defects.^[^ ^10^, ^19^, ^20^, ^21^, ^22^ ^]^ However, rheology only provides averaged macroscopic properties and is not able to quantitatively measure nanostructural defects. To quantify these defects, low‐field time‐domain ^1^H NMR relaxometry (TD‐NMR) proved to be a versatile non‐invasive characterization technique.^[^ ^23^, ^24^, ^25^, ^26^, ^27^ ^]^ This method probes the polymer segmental mobility (<10 nm) from which nanoscopic structural aspects of the network can be inferred. For instance, through the application of spin echo pulse sequences, the segmental mobility of polymer chains can be assessed by the transverse relaxation time *T*_2_.^[^ ^25^, ^28^, ^29^, ^30^ ^]^

A direct quantitative correlation of both the mechanical properties and the segmental mobility is challenging due to inherent differences in the experimental design, varying sample preparation, and reaction conditions. To enable a direct in situ correlation, we developed a unique combined low‐field RheoNMR setup that was previously used to investigate the shear‐induced crystallization kinetics of isotactic poly(propylene). In this approach, we used TD‐NMR to measure the crystallinity based on *T*_2_ relaxation curves and monitored *G*′ as a function of crystallinity.^[^ ^31^, ^32^ ^]^ The apparatus consists of a portable low‐field ^1^H NMR unit (25 MHz Larmor frequency) that is attached to a commercial high‐end rheometer.

**Figure 1:**
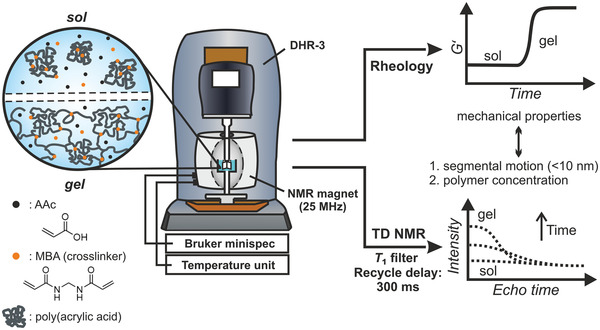
Schematic of the combined RheoNMR setup consisting of a ^1^H NMR unit (25 MHz Larmor frequency) that is implemented in a stress‐controlled rheometer (DHR‐3) and the respective experimental observables: elastic shear modulus *G*′ and *T*_2_ relaxation curve. The combined setup is used to monitor the gelation process from the sol (liquid) to gel (solid) state during the free radical crosslinking copolymerization of AAc and MBA.

Here, we use the RheoNMR setup to examine whether macroscopic mechanical properties of PAAc gels are reflected in the molecular motion (<10 nm) by obtaining a unique in situ correlation of *G*′ with *T*_2_ relaxation times. Our aim is to study the impact of varying crosslinker concentrations on mechanical properties while simultaneously measuring network formation via TD‐NMR. Despite the importance of this type of hydrogel in several applications, such a correlation has to the best of our knowledge not been investigated. An explanation for this gap could be the challenging free radical reaction mechanism that exhibits rapid gelation kinetics, distinctly increasing measurement complexity in both rheology and TD‐NMR. Hence, we discuss in detail the experimental parameters used in the rheological and TD‐NMR measurements. We further use TD‐NMR as a concentration probe to correlate *G*′ with the polymer concentration during gelation by applying a *T*_1_ filter that suppresses NMR signal intensity of monomer and solvent. The RheoNMR setup and the respective rheological and TD‐NMR observables are shown in **Figure** **1**. A photograph of the setup is shown in Figure S1, Supporting Information.

## 2 Experimental Section

### 2.1 Materials

2,2'‐Azobis[2‐(2‐imidazolin‐2‐yl)propane]dihydrochloride (VA‐044, 95%, FUJIFILM Wako Pure Chemical), deuterium oxide (D_2_O, 99 %, Sigma‐Aldrich) and *N*,*N*'‐methylene bisacrylamide (MBA, 99 %, Sigma‐Aldrich) were used as received. Acrylic acid (AAc, > 99 %, Merck) was freshly distilled at reduced pressure prior to the synthesis.

### 2.2 Sample Preparation

PAAc hydrogels were synthesized by aqueous free radical copolymerization of AAc and MBA. The weight fraction of the initiator VA‐044 to AAc was 0.5 wt%. The total monomer weight fraction in D_2_O (solvent) was kept constant to 20 wt%. The degree of crosslinking (*DC*) defined as the molar ratio of MBA to AAc was varied to target the following values: *DC* = 0, 0.05, 0.1, 0.2, 0.5, and 1 mol%. In the following, the preparation of the sample with *DC* = 1 mol% is described in detail as an example. First, MBA (77.0 mg, 0.5 mmol) was dissolved in D_2_O (12.3 mL) and subsequently freshly distilled AAc (3.6 g, 50.0 mmol) was added to the reaction mixture. The initiator VA‐044 (18.0 mg, 0.06 mmol) was separately dissolved in D_2_O (2 mL) and added to the mixture. The pre‐gel solution was cooled to 0 °C, stirred vigorously for 1 min and then 0.8 mL of the solution was poured into the lower DHR‐3 cup geometry.

### 2.3 Apparatus

The low‐field RheoNMR setup consists of a portable ^1^H NMR unit that was attached to a commercial DHR‐3 rheometer (TA Instruments). The NMR magnet was based on a Halbach array of NdFeB permanent magnets (*B*_0_ = 0.6 T, *ω*_L_/2*π* = 25 MHz for ^1^H). Detailed description of the construction and setup has been described previously.^[^ ^31^ ^]^ The employed NMR probe has a dead time of 10 *µ*s and pulse lengths of 2.2 *µ*s (90°) and 4.4 *µ*s (180°). Data acquisition and pulsing were performed on the Bruker “the minispec” electronic unit (NF series). The temperature was controlled using a Bruker VTU unit to 40 °C at an air flow rate of 270 L h^−1^.

### 2.4 Rheological Measurements

To ensure reproducibility of the rheological measurements throughout the whole sample range, wall‐slip beyond the gel point has to be avoided. Therefore, strain‐controlled measurements at a low strain of 0.5 % and a vane‐cup geometry were used as the method of choice to monitor the structural build‐up of the gel.^[^ ^33^, ^34^, ^35^ ^]^ The oscillatory shear experiments were performed using a vane geometry (4 blades with diameter: 8 mm, height: 11 mm, width: 1.2 mm) and a cup (diameter: 11 mm, height: 13 mm) that were made out of proton‐free poly(chlorotrifluoroethylene) (PCTFE). The distance from the vane geometry to the bottom of the cup was set to 1 mm. The gelation was monitored using an oscillatory time sweep at a constant nominal strain of 0.5 % and an angular frequency of 6.8 rad s^−1^, which was found to be in the linear viscoelastic regime of a gelled sample by conducting a strain sweep from 0.5 % to 300 % at 6.8 rad s^−1^. The nominal strain was calculated in the rheometer software by multiplying the implemented standard geometry strain constant of a double wall concentric cylinder with the motor angular displacement.

### 2.5 Time‐Domain ^1^H NMR Measurements

**Figure 2:**
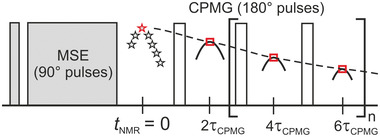
Combined MSE‐CPMG pulse sequence used to measure the transverse magnetization decay with 2*τ*_cpmg_ = 0.1 ms and *n* = 255. Red symbols mark the echo maxima that are used for evaluation. An XX4 phase cycle in the CPMG train is used to avoid spin locking effects.^[^ ^38^ ^]^ The MSE echo maximum defines the signal intensity at *t*_NMR_ = 0 ms.^[^ ^39^ ^]^

The segmental mobility during gelation was monitored by ^1^H *T*_2_ relaxation measurements using a combination of an MSE (magic sandwich echo) and CPMG (Carr, Purcell, Meiboom, Gill) pulse sequence, as shown in **Figure** **2**.^[^ ^36^, ^37^ ^]^ The CPMG sequence uses an XX4 phase cycle to avoid spin locking effects.^[^ ^38^ ^]^ The MSE measures the transverse magnetization over the dead time (10 *µ*s) after a 90° pulse and was used to determine the initial signal intensity at *t*_NMR_ = 0 ms.^[^ ^39^ ^]^ The CPMG sequence (512 echoes) refocuses the transverse magnetization of the sample with a delay of 2*τ*_CPMG_ = 100 *µ*s between subsequent 180° pulses.

**Figure 3:**
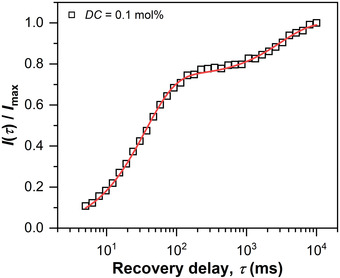
Build‐up of the longitudinal magnetization of a fully polymerized sample with *DC* = 0.1 mol% measured by an SR measurement. The solid line represents a least‐squares fit using Equation (1).

The pulse sequence (see Figure 2) ends with a recycle delay (RD) of 300 ms and 8 scans were accumulated for signal‐averaging, which leads to a duration of 5 s per experiment. This choice ensures a sufficient time resolution for monitoring polymerization kinetics. The longitudinal relaxation *T*_1_ time was measured using a saturation recovery (SR) pulse sequence, as shown in **Figure** **3** for a fully polymerized sample with *DC* = 0.1 mol%. The SR build‐up curve was evaluated by a biexponential fit

(1) $$\begin{array}{c} \begin{array}{c} I\left(\tau \right)=A_{\mathrm{short}}\;\left[1-\exp\left(-\frac{\tau }{T_{1,\mathrm{short}}}\right)\right]+A_{\mathrm{long}}\left[1-\exp\left(-\frac{\tau }{T_{1,\mathrm{long}}}\right)\right] \end{array} \end{array}$$

where *A*_short _+ *A*_long_ = 1 and *T*_1_ is the longitudinal relaxation time in the order of milliseconds (short) and seconds (long).

Two components with *T*_1,long_ = 3 s and *T*_1,short_ = 35 ms were found, which were assigned to the solvent (residual HDO and monomer) and polymer proton signal, respectively. The fitting results are summarized in **Table** **1**.

**Table 1:** SR build‐up curve analysis using Equation (1) of the sample *DC* = 0.1 mol% with fractions A of the short and long component and the corresponding *T*_1_ times. The results are assigned to polymer proton signal and solvent. The obtained polymer to solvent fraction ratio of approximately 3:1 is consistent with the chemical structure of PAAc, considering three protons in the polymer backbone and the one protic hydrogen atom of the carboxyl group that undergoes a hydrogen–deuterium exchange with D_2_O

Assignment	*A*	*T*_1_ [ms]	Description
Short	0.74 ± 0.01	35 ± 0.5	Crosslinked, entangled or linear polymer chains
Long	0.26 ± 0.01	3000 ± 260	HDO or residual monomer

According to the SR build‐up curve, a RD of 300 ms is larger than five times the *T*_1_ of the polymeric species. Consequently, the chosen RD functions as a *T*_1_ filter that suppresses the signal of the solvent to approximately 1‐exp(−300/3000) ≈ 10 % of the maximum intensity. Note that the longitudinal relaxation was affected by high‐frequency motions in the order of the Larmor frequency (25 MHz) and was not significantly influenced by weak constraints of the crosslinks in the polymer network. The SR measurement was independent of *DC*, as shown in Figure S2, Supporting Information, and the chosen RD applicable in the whole sample range.

The combined measurements were repeated three times for every gel composition. The data points in the respective graphs were the mean values with error bars representing 1 standard deviation.

## 3 Results and Discussion

### 3.1 Gelation Kinetics Measured by Oscillatory Shear Rheology

**Figure 4:**
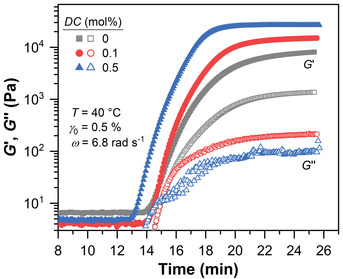
Time‐evolution of the elastic *G*′ and loss modulus *G*′′ during the free radical crosslinking copolymerization of AAc and MBA with varying *DC* values. The mechanical response has three characteristic phases: an induction period in the first 13–14 min, a rapid increase of the moduli by three magnitudes beyond the gel point, and an approach to a plateau value.

The mechanical response of the free radical crosslinking copolymerization of AAc and MBA was monitored by an oscillatory time sweep experiment. The time‐evolution of the elastic *G*′ and loss modulus *G*′′ is shown in **Figure** **4**. All samples show a similar sigmoidal curve with three characteristic phases. After a certain induction phase of approximately 14 min, *G*′ and *G*′′ increase rapidly by 1 to 3 orders of magnitude and reach a plateau value. The measured *G*′ value of ≈5 Pa in the induction phase results from the intrinsic inertia of the vane geometry due to the low viscosity of the pre‐gel solution that is below the instrument sensitivity (≈1.7 *μ*Nm) for our setup.

**Figure 5:**
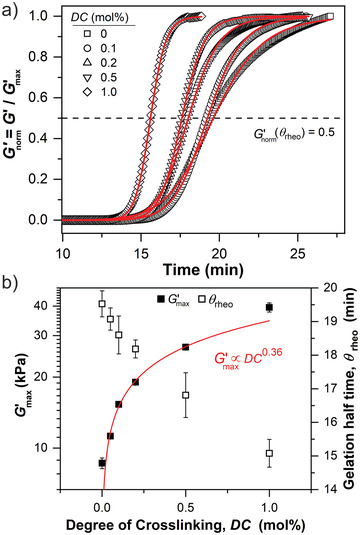
a) Normalized time‐evolution of the elastic modulus *G*′ during the gelation of AAc for varying *DC* values. The solid lines represent a least‐squares fit using Equation (2) with *θ*_rheo_ as the gelation half time. b) Maximum elastic modulus $G_{max}^{\prime }$ and *θ*_*rheo*_ as a function of *DC*. The solid line represents a least‐squares fit using a simple power law (scaling exponent = 0.36 ± 0.02; prefactor = 35 kPa mol%^−0.36^).

To investigate the impact of *DC* on the gelation kinetics, we normalized *G*′ to the maximum value, *G*′_norm_ = *G*′/*G*′_max _, and evaluated the time‐evolution of $G_{\mathrm{norm}}^{\prime }$ by

(2) $$G_{\mathrm{norm}}^{\prime }\left(t\right)=\frac{t^{n}}{t^{n}+\theta_{\mathrm{rheo}}^{n}}$$

where *θ*_rheo_ is the rheological gelation half time with $G_{\mathrm{norm}}^{\prime }\;\left(\theta_{\mathrm{rheo}}\right)=\;0.5$ and n is the gelation rate exponent, which is proportional to the slope at *t* = *θ*_rheo_ according to $\dot{G}\left(\theta_{\mathrm{rheo}}\right)\;=\frac{n}{4\theta_{\mathrm{rheo}}}$.^[^ ^15^, ^16^, ^18^ ^]^ The normalized mechanical responses during gelation of AAc with varying *DC* values and the corresponding fits are shown in **Figure** **5**a. The dependency of $G_{\max}^{\prime }$ and *θ*_rheo_ on *DC* is shown in Figure 5b.

The amount of crosslinker in the pre‐gel solution distinctly influences the gelation behavior. The gelation half time *θ*_rheo_ decreases from 19 min for a slightly crosslinked sample with *DC* = 0.05 mol% to 15 min at *DC* = 1 mol%. The respective $G_{\max}^{\prime }$ values increase from 11 kPa to 40 kPa. A simple power law with a scaling exponent of 0.36 describes the dependency of $G_{\max}^{\prime }$ on *DC*. This scaling, however, deviates from the expected linear relationship of $G_{\max}^{\prime }$ and *DC* based on the rubber elasticity theory, indicating a decrease of crosslinking efficiency at increasing MBA concentrations.^[^ ^40^, ^41^ ^]^

Note that we kept the length of the time sweep constant at around 25 min to avoid a steady increase of the elastic plateau modulus due to simple water evaporation. Thus, the plateau of *G*′ for loosely crosslinked samples with *DC* = 0 and 0.05 mol% is not fully reached, which is in the margin of error in the rheological measurements. In addition, despite the use of a low strain of 0.5%, we observed wall slips (not shown) for the highest crosslinked sample with *DC* = 1 mol% where *G*′ rapidly dropped by approximately 10 % due to a contraction of the material. Hence, we cut the data points beyond the wall slip for the evaluation of the gelation kinetics (see Figure 5a). This does not influence the overall kinetics as clearly a plateau value had been reached at this point.

**Figure 6:**
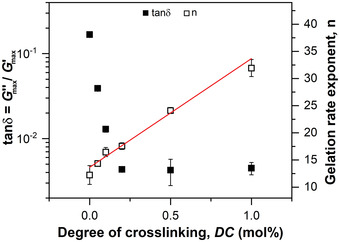
The loss tangent tan*δ* = $G_{max}^{{}^{\prime \prime }}$/$\;G_{max}^{\prime }$ and the gelation rate exponent n, which is obtained by Equation (2), as a function of *DC*. The solid line represents the result of a linear regression analysis (slope = 20 mol%^−1^; intercept = 14; *R* ^2^ = 0.99).

The influence of *DC* on the final loss tangent tan*δ* = *G*′′_max _/*G*′_max _, which gives the ratio of the viscous dissipation to the elastic response, and the gelation rate exponent n is shown in **Figure** **6**.

The loss tangent decreases by two orders of magnitude as a function of *DC* from tan *δ* = 0.2 for PAAc at *DC* = 0 mol% to tan*δ* = 0.004 at *DC* =1 mol%, ultimately reaching a plateau value that corresponds to the limit in sensitivity of the setup. This confirms gel formation throughout the sample range. At *DC* = 0 mol% a semi‐dilute entangled polymer solution is formed where physical rather than chemical crosslinks are present. An increase of *DC* values further introduces covalent crosslinks, distinctly enhancing the elastic response and the gelation rate. The gelation rate exponent n is in the range of 12 to 32. This high value reflects the fast gelation kinetics of the free radical crosslinking copolymerization reaction. The gelation rate exponent n increases linearly as a function of *DC*. Hence, the concentration of MBA is rate determining for the gelation, yet simultaneously a reduction of the crosslinking efficiency at higher *DC* values is observed (see Figure 5b). This loss in efficiency suggests that inelastic defects are formed during gelation. To elucidate the origin for this defect formation based on a molecular explanation of the mechanical properties, we discuss TD‐NMR measurements in the following.

### 3.2 Nanostructural Insights into the Gelation Process via TD‐NMR

Rheology probes the macroscopic averaged properties and is not able to provide quantitative molecular insight into the network structure. As both mechanical properties and polymer segmental mobility undergo a rather fast time‐evolution during gelation, any separate measurements will be of limited accuracy towards a direct correlation of both quantities. We use the on‐line combination of advanced rheology with low‐field TD‐NMR to overcome this limitation. The CPMG/XX4 echo train (see Figure 2) acquires the *T*_2_ relaxation curves, which are directly linked to the segmental motion, and therefore, are related to nanoscopic (<10 nm) topological constraints.^[^ ^25^ ^]^ We find that a stretched exponential (Kohlrausch–Williams–Watts) function describes the *T*_2_ relaxation curves well throughout the gelation process:

(3) $$I\left(t_{\mathrm{NMR}}\right)=\;A\;\text{exp}\left(-{\left(\frac{t_{\mathrm{NMR}}}{T_{2}}\right)}^{\beta }\right)+\mathrm{offset}$$

**Figure 7:**
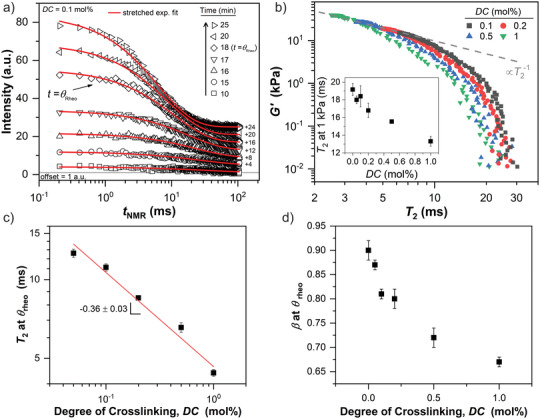
a) Transverse relaxation *T*_2_ curves measured by the MSE‐CPMG/XX4 pulse sequence at different gelation times in the range of 10 min (sol) to 25 min (fully crosslinked). The solid lines represent least‐squares fits using a stretched exponential function (see Equation (3)). For better visibility, *T*_2_ relaxation curves are shifted upwards in steps of 4 a.u. b) Correlation of *G*′ with *T*_2_ relaxation times for varying *DC* values, displayed on a log‐log‐scale. The inset shows *T*_2_ at 1 kPa as a function of *DC*. c) The *T*_2_ relaxation times at *θ*_rheo_ as a function of *DC*, displayed on a log‐log scale. The solid line represents a least‐squares fit according to a simple power law (scaling exponent = −0.36 ± 0.03; prefactor = 4.7 ± 0.3 ms mol%^0.36^). d) The stretching exponent *β* at *θ*_rheo_ as a function of *DC*. A reduction of *β* indicates the increasing formation of nanoscopic defects with a broad distribution of *T*_2_ relaxation times.

*T*_2_ is the transverse relaxation time of the polymer network, *β* < 1 is the stretching exponent and the offset is set to 1 a.u.^[^ ^42^, ^43^, ^44^ ^]^ **Figure** **7** shows the time‐evolution of the *T*_2_ relaxation curves, the obtained results of the stretched exponential fits, and the dependency of *G*′ on *T*_2_ times.

As the crosslinking copolymerization proceeds, the sample transitions from the liquid sol state to the solid gel state after an induction period of approximately 13 min. Correspondingly, in the sol state, a low initial signal intensity of 6 a.u. is observed. This low signal intensity is residual solvent signal, which is substantially suppressed by the *T*_1_ filter. Beyond the gel point, indicated by a distinct increase in *G*′, the polymer network is formed (see Figure 5a). Consequently, the signal intensity increases by almost a factor of 10 to a maximum of *I*_max_ = 56 a.u. as more polymer chains with a *T*_1_ below the *T*_1_ filter are formed.

Figure 7b shows *G*′ as a function of *T*_2_ times. The *T*_2_ relaxation time is connected to the nanoscopic segmental mobility (<10 nm) of the polymer chains via the orientation‐dependent homonuclear dipolar couplings of neighboring ^1^H spins along the polymer backbone.^[^ ^23^, ^24^, ^25^, ^29^, ^45^, ^46^ ^]^ In isotropic not entangled solutions this interaction is fast and time‐averaged to zero on the NMR time scale, and therefore, not observable. The presence of chemical and physical crosslinks in polymer networks prevents this motional averaging and a residual homonuclear dipolar coupling is observed. The strength of this dipolar coupling is reflected in the *T*_2_ relaxation time. For instance, the mobility of shorter network chains is more restricted, which increases the anisotropy of segmental motion, and therefore, enhances spin‐spin interactions, leading to lower *T*_2_ relaxation times.

The correlation of *G*′ with *T*_2_ times follows a characteristic trend. In the beginning of the gelation at low *G*′ values ranging from 0.1 to 1 kPa, a constant *T*_2_ time in the range of 20 to 30 ms is observed. At early gelation times, a loosely crosslinked network is formed that consists of highly mobile chains. The segmental motion of those mobile chains is not influenced by the crosslinks, and therefore, the *T*_2_ time is independent of the average crosslink density measured by rheology. At higher *G*′ values above 1 kPa, the curve approaches an inverse relationship ($G^{\prime }\propto T_{2}^{-1}$). Hence, higher crosslink densities restrict the mobility of polymer chains between crosslinks, enhancing the residual dipolar coupling. The inverse relationship further shows that the mechanical properties are similarly reflected in the *T*_2_ times, which depend on the average molecular weight of network strands (i.e., crosslink density). This finding is in agreement with previous *T*_2_ relaxation studies of rubbery materials where the *T*_2_ time has been correlated to the conformational mean position of polymer chain segments between crosslinks.^[^ ^30^, ^47^, ^48^, ^49^, ^50^ ^]^

Moreover, a shift of *T*_2_ times towards lower values is observed for increasing *DC*. This shift suggests that increasing crosslinker concentrations induce an initial molecular stiffness without affecting *G*′. This is further shown in the inset of Figure 7b where *T*_2_ at *G*′ = 1 kPa decreases linearly as a function of *DC*. We attribute this early molecular stiffness at higher *DC* to the formation of inelastic, rigid domains such as crosslinking cluster. To further investigate the influence of *DC* on the network structure, we use *θ*_rheo_ as a reference point during gelation. The dependencies of *T*_2_ times and *β* at *θ*_rheo_ on *DC* are shown in Figure 7c,d, respectively. The *T*_2_ time decreases with a scaling exponent of −0.36 as a function of *DC* from 13 ms at a low *DC* = 0.05 mol% to 4 ms at *DC* = 1 mol%. Considering the inverse relationship of *T*_2_ with $G_{\max}^{\prime }$, this scaling is identical with the rheological data and clearly demonstrates that the macroscopic mechanical properties, such as the loss of crosslinking efficiency at higher *DC* values (see Figure 5b), are similarly reflected in the nanoscopic segmental mobility of the material.

The stretching exponent *β* decreases as a function of *DC* from *β* = 0.90 at *DC* = 0 mol% to *β* = 0.67 at *DC* = 1 mol%. For more homogenous and uncharged networks such as vulcanized rubbers, the transverse magnetization decay is typically rather well described by a compressed exponential where *β* is in the range of 1 to 2.^[^ ^28^, ^51^ ^]^ A deviation from this behavior in the form of a stretched exponential is characteristic for heterogeneous systems on the molecular level where the stretching exponent *β* (see Equation (3)) reflects the superposition of dynamically different topologies.^[^ ^24^, ^25^, ^52^ ^]^ Hence, the *T*_2_ relaxation curve in heterogeneous systems is a weighted sum of exponential decays associated with different network chains such as loops, and dangling ends. To be more quantitative, *β* ^−1^ is related to the width of the distribution function.^[^ ^53^ ^]^ Therefore, lower *β* values indicate the formation of a more heterogeneous sample with a broader distribution of *T*_2_ times. Without the addition of crosslinker, the value of *β* = 0.9 can be attributed to the intrinsically complex free radical polymerization mechanism that causes branching via intramolecular chain transfer reactions.^[^ ^54^, ^55^, ^56^, ^57^ ^]^ The additional decrease of *β* with increasing *DC* suggests that MBA distinctly enhances the formation of nanoscopic inhomogeneities as reflected by a broad distribution of *T*_2_ times. At higher MBA concentrations the probability for the formation of inelastic intramolecular crosslinks increases, for instance, due to additional pendant vinyl bonds of the crosslinker along the polymer backbone that allows for cyclization reactions. This formation of intramolecular crosslinks is presumably further enhanced by unequal copolymerization parameters of AAc and MBA as well as the intrinsic high reactivity of MBA because of the divinyl functionality.^[^ ^58^ ^]^

These results are in agreement with previous kinetic studies of the free radical crosslinking copolymerization of MBA with acryl amide where a loss in crosslinking efficiency was attributed to the formation of intramolecular crosslinks in the form of microgels, which was confirmed by light scattering techniques and the analysis of pendant vinyl bond conversion.^[^ ^9^, ^20^, ^52^ ^]^ This should be considered when MBA is used to increase the mechanical strength of the gels since properties such as fracture resistance and transparency are highly affected by the so‐called local inhomogeneities.^[^ ^10^, ^11^ ^]^ This finding is consistent with the rheological and *T*_2_ data that both show a decrease in the crosslinking efficiency at higher *DC* values, which can be associated with the formation of inelastic network defects on the molecular level and quantified by *β*.

### 3.3 Correlating the Elastic Modulus with Polymer Concentration

**Figure 8:**
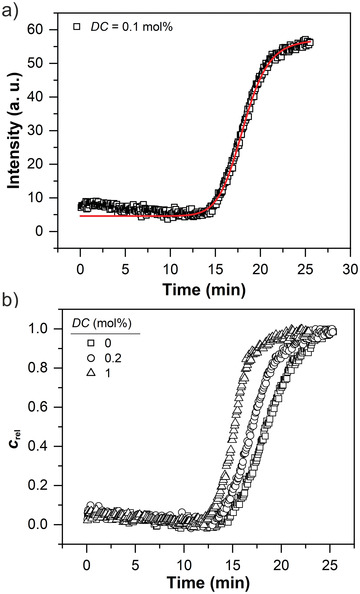
a) Time‐evolution of the initial (*t*_NMR_ = 0 ms) NMR signal intensity during gelation. The solid line represents a least‐squares fit using Equation (4) in the range of 10 to 25 min. b) Time‐evolution of the relative polymer concentration defined as $c_{\mathrm{rel}}=\frac{I-I_{\mathrm{solv}}}{I_{\max}-I_{\mathrm{solv}}}\;$for varying *DC* values. The characteristic sigmoidal curve behavior is in agreement with the time‐evolution of the elastic response.

To fully understand the mechanical response during gelation, the time‐evolution of *G*′ can be directly correlated to the polymer concentration during the copolymerization. TD‐NMR is intrinsically quantitative as the signal intensity is proportional to the number of ^1^H nuclear spins. Consequently, it can be used to probe the time‐evolution of the polymer concentration. Here, we use a *T*_1_ filter to suppress the contribution of solvent to the NMR signal intensity. The increase of the NMR signal intensity during gelation can then be associated with a relative polymer concentration *c*_rel_. To achieve this, we quantify residual solvent signal by evaluating the time‐evolution of the initial (*t*_NMR_ = 0 ms) NMR signal intensity using

(4) $$I\left(t\right)=\frac{\left(I_{\max}-I_{\mathrm{solv}}\right)t^{m}}{t^{m}+\theta_{\mathrm{NMR}}^{m}}\;+I_{\mathrm{solv}}$$

where *I*_max_ is the maximum signal intensity, *I*_solv_ is the residual signal intensity of the solvent, *θ*_NMR_ is the NMR gelation half time and m is the gelation rate exponent obtained from NMR data. To isolate the contribution of polymer to the signal intensity, we subtracted the solvent signal *I*_solv_ from the raw data and normalized the signal intensity to the maximum value. The relative polymer concentration is defined as $c_{\mathrm{rel}}=\frac{I-I_{\mathrm{solv}}}{I_{\max}-I_{\mathrm{solv}}}$. **Figure** **8**a shows the time‐evolution and evaluation of the NMR signal intensity for the sample with *DC* = 0.1 mol%.

As the polymerization proceeds, *c*_rel_ increases as a function of time and reaches a plateau value, mirroring the underlying sigmoidal curve characteristics of the rheological data. An overview of all the gelation kinetic parameters obtained by NMR and rheology is shown in Table S1, Supporting Information. A slight decrease of signal intensity from 0.10 to 0.06 a.u. is observed during the first 10 min of the gelation (see Figure 8a). We attribute this early decay of the signal intensity to the consumption of oxygen, which is known to shift the *T*_1_ time of the environment to lower values, by initiator radicals. To strengthen this argument, we studied the time‐evolution of the NMR signal intensity of a degassed sample (two freeze‐pump‐thaw cycles), which does not show such a decay, as shown in Figure S3, Supporting Information.

**Figure 9:**
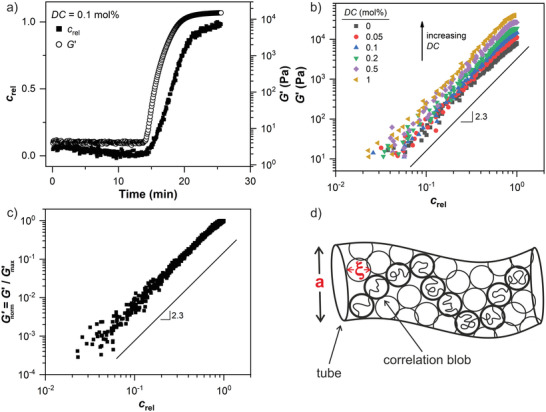
a) Time‐evolution of the relative polymer concentration *c*_rel_ and *G*′ for the sample with *DC* = 0.1 mol%. b) Plot of *G*′ as a function of *c*_rel_ during the gelation for varying *DC* values, displayed on a log‐log scale. The prefactor corresponds to $G_{\max}^{\prime }$ and its dependency on *DC* is shown in Figure 5b. c) Normalized *G*′ as a function of *c*_rel_ throughout the whole sample range. The solid line represents a characteristic scaling law ($G_{\mathrm{norm}}^{\prime }$ = $c_{\mathrm{rel}}^{2.3}$) based on the confining tube model in semi‐dilute entangled polymer solutions. d) Confining tube of a polymer chain (thick cycles) in a semi‐dilute entangled polymer solution.^[^ ^59^, ^60^, ^61^ ^]^ The entanglement strand is described by a random walk of correlation blobs with size *ξ* inside a tube with diameter *a*. Thin cycles represent correlation blobs of neighboring polymer chains. The points of contact between cycles are associated with chemical and physical crosslinking points.

The correlation of *G*′ with *c*_rel_ is shown in **Figure** **9**. We find that the correlation follows a characteristic scaling law ($G^{\prime }\propto c_{\mathrm{rel}}^{2.3}$) that is independent of *DC*.

The theoretical prediction of the polymer concentration dependency of *G*′ is based on the confining tube model in semi‐dilute entangled polymer solutions that considers two characteristic length scales.^[^ ^59^, ^60^, ^61^, ^62^ ^]^ The correlation length *ξ*, which describes the distance to the neighboring chains (i.e., mesh‐size), and the Edwards tube diameter (*a* > *ξ*). The elastic response is described by a random walk of blobs with a diameter *ξ* inside the tube with diameter *a* that covers the entanglement strands.^[^ ^61^ ^]^ Hence, in agreement with the rubber elasticity theory,^[^ ^63^ ^]^ where the elastic modulus equals the number density of entanglement strands times the thermal energy *kT*, *G*′ can be expressed as a function of *a* and *ξ*  that define the entanglement volume.^[^ ^59^, ^60^, ^61^, ^62^ ^]^ The parameters *a* and *ξ* are independent of the molecular weight and scale with the polymer concentration *c* in semi‐dilute entangled polymer solutions. This leads to the following relation of *G*′ with the polymer concentration

(5) $$G{}^{\prime }\;=\frac{kT}{a^{2}\xi }\;\propto \;c^{k}$$

where the exponent *k* is equal to 2.33 in a theta‐solvent and 2.31 in a good solvent.^[^ ^59^, ^60^ ^]^ A schematic of this confining tube model is shown in Figure 9d.

The experimentally obtained scaling $G^{\prime }\propto c_{\mathrm{rel}}^{2.3}$ during gelation of AAc is in agreement with the theoretical prediction of *G*′ and in the margin of error independent of *DC*. At higher *DC* values more elastic chains per volume are introduced into the polymer network, reducing the correlation length *ξ* between neighboring polymer chains and the tube diameter *a* without affecting the concentration‐dependent scaling laws. Hence, despite the rapid polymerization kinetics, the RheoNMR approach can be used to correlate *G*′ with *c*_rel_ by applying a *T*_1_ filter that suppresses solvent signal. In future studies, we will use this approach as an alternative method to probe theoretical predictions of *G*′ for different experimental conditions, such as theta‐solvent, and polyelectrolytes.

To conclude the RheoNMR findings, the time‐evolution (*t* in min) of *G*′ (kPa) during AAc crosslinking copolymerization can be expressed as a function of both the synthetic parameter *DC* (mol%) and the dependency on *c*_rel_ (dimensionless) by

(6) $$G^{\prime }=G_{\max}^{{}^{\prime }}\;c_{\mathrm{rel}}^{2.3}$$

and

(7) $$G^{\prime }=35\;DC^{0.36}\;{\left(\frac{t^{m}}{t^{m}+\theta_{\mathrm{NMR}}^{m}}\right)}^{2.3}$$

The first term of Equation (7) describes the impact of crosslinker concentration in the pre‐gel solution on the elastic plateau modulus ($G_{\max}^{\prime }=\;35DC^{0.36}$), as shown in Figure 5b, from which the final crosslinking efficiency can be inferred. The second term describes the gelation kinetics as function of time obtained by TD‐NMR.

## 4 Conclusion

To further understand the relation between macroscopic mechanical and molecular properties in hydrogel synthesis, we present a unique characterization method based on the combination of oscillatory shear rheology and low‐field TD‐NMR relaxometry, referred to as low‐field RheoNMR. While rheology measures the macroscopic mechanical properties, TD‐NMR probes the *T*_2_ relaxation curves to obtain a molecular insight into the network structure as an additional information to the rheological data. We use this approach to monitor the gelation during the free radical crosslinking copolymerization of AAc and MBA at varying *DC*. We find that the *T*_2_ relaxation of the ^1^H NMR magnetization of the gels can be rather well described by a stretched exponential function throughout the gelation process. The stretching exponent decreases as a function of *DC* from 0.9 at *DC* = 0 mol% to 0.67 at *DC* = 1 mol%. Since the inverse of the stretching exponent is related to the width of the *T*_2_ distribution, this decrease indicates the formation of a heterogeneous network with dynamically different topologies at increasing *DC*. The direct in situ inverse correlation of *G*′ with *T*_2_ times further shows that higher *DC* values increase molecular stiffness at early gelation times without affecting *G*′, suggesting the formation of inelastic, rigid domains such as crosslinking clusters. Moreover, we use TD‐NMR to correlate *G*′ with the polymer concentration. Beyond the gel point, we find a characteristic scaling exponent of 2.3, which is in agreement with theoretical predictions of *G*′ by means of polymer dynamics in semi‐dilute entangled polymer solutions. In future studies, we will use this approach to investigate the effect of theta conditions and polyelectrolytes with regards to the theoretical predictions.

## Conflict of Interest

The authors declare no conflict of interest.

## Supporting information

Supporting Information

## Data Availability

The data that support the findings of this study are available from the corresponding author upon reasonable request.
