# Supplementary material for: In Situ RheoNMR Correlation of Polymer Segmental Mobility with Mechanical Properties during Hydrogel Synthesis
Source: Adv Sci (Weinh). 2021 Dec 11;9(4):2104231. doi: 10.1002/advs.202104231 (PMC8811812; doi:10.1002/advs.202104231)
Supplement: Supplementary file 1 — Supporting Information [file ADVS-9-2104231-s001.pdf]

ADVANCED SCIENCE
Open Access

Supporting Information

for *Adv. Sci.*, DOI: 10.1002/advs.202104231

In-situ RheoNMR correlation of polymer segmental mobility with mechanical properties during hydrogel synthesis

*Christian Fengler, Jonas Keller, Karl-Friedrich Ratzsch and Manfred Wilhelm**

Supporting Information

In-situ RheoNMR correlation of polymer segmental mobility with mechanical properties during hydrogel synthesis

*Christian Fengler, Jonas Keller, Karl-Friedrich Ratzsch and Manfred Wilhelm**

* E-mail: manfred.wilhelm@kit.edu.

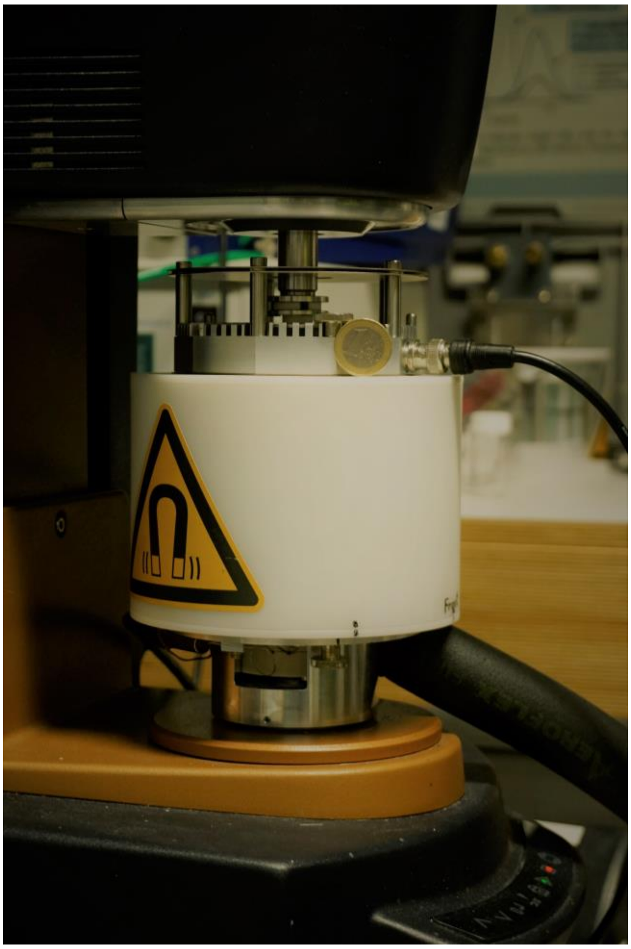

Figure S1. Photograph of the RheoNMR setup. The NMR magnet is attached to a DHR-3 rheometer.

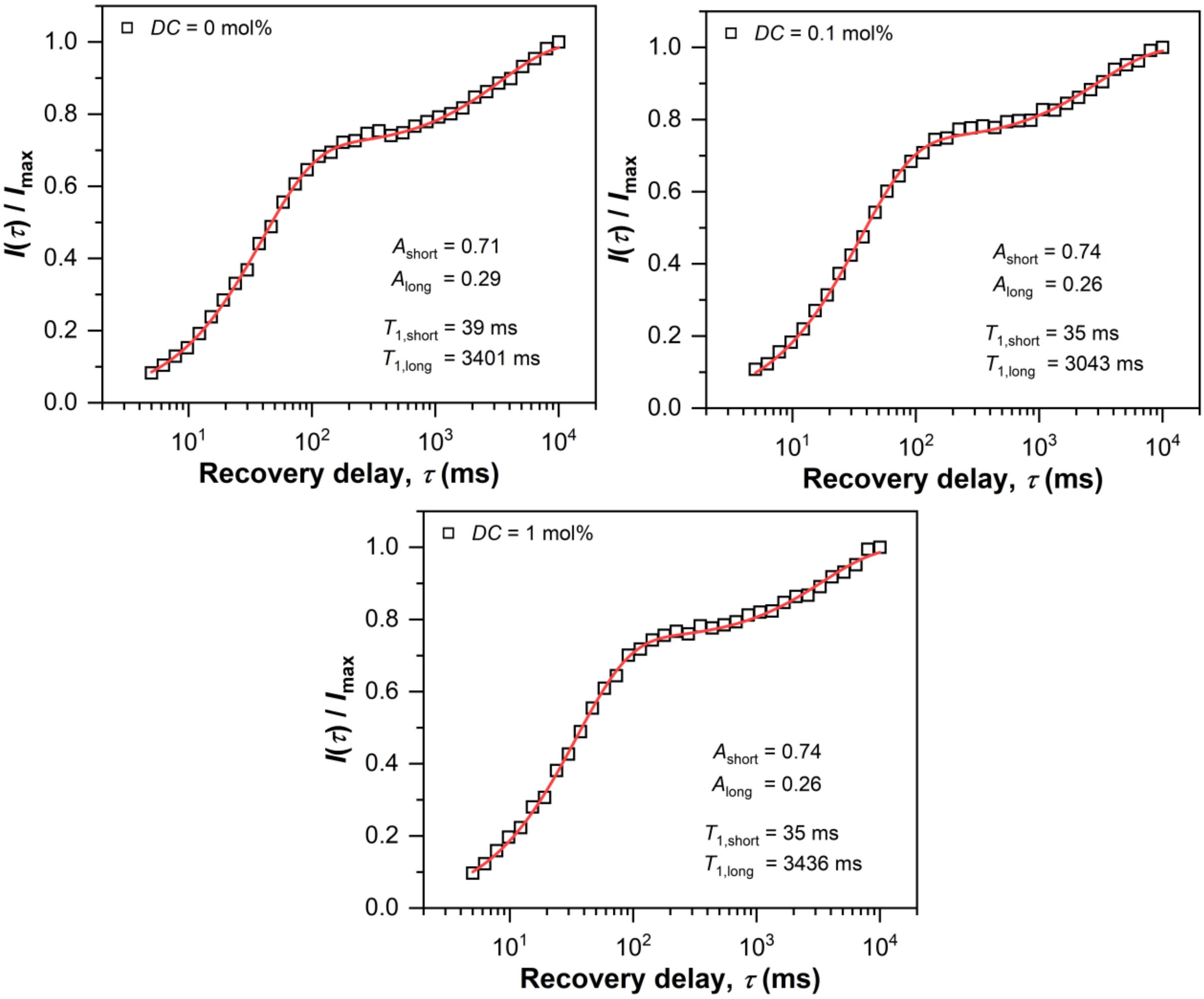

Figure S2. Saturation recovery (SR) experiments for three different PAAc gels with varying degree of crosslinking *DC* in the range of 0 to 1 mol% to determine the longitudinal relaxation time T_1. The solid lines represent least-squares fits using Equation 1. The $T_{1,short}$ of the polymer is in the margin of error independent of *DC* and shows that the chosen recycle delay of 300 ms is applicable in the whole sample range.

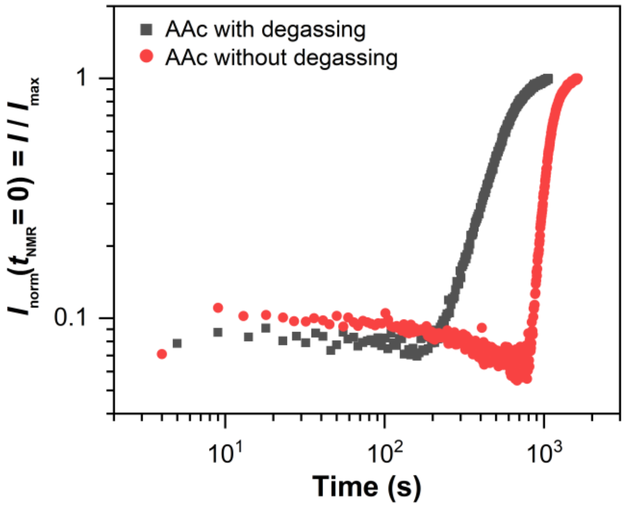

Figure S3. Evolution of the ^{1}H NMR signal intensity during gelation of a degassed (two freeze-pump-thaw cycles) and non-degassed acrylic acid sample with DC = 0 mol%. The degassed sample does not show a drop in signal intensity compared to the non-degassed sample, which is attributed to the consumption of oxygen by the initiator radicals.

Table S1. Overview of the gelation kinetic parameters for samples with varying degrees of crosslinking DC measured by the time-evolution of G' (rheology) and NMR signal intensity using Equation 2 and Equation 4, respectively. θ_{rheo} is the rheological gelation half time, n is the rheological gelation rate exponent, θ_{NMR} is the NMR gelation half time, m is the NMR gelation rate exponent, G'_{max} is the maximum value of the elastic modulus, I_{max} is the maximum NMR signal intensity and I_{solv} is the residual solvent NMR signal intensity.

***DC* (mol%)**	**G'_{max} (kPa)**	**θ_{rheo} (min)**	**n**	**I_{max} (a.u.)**	**θ_{NMR} (min)**	**m**	**I_{solv} (a.u.)**
**0**	8.6 ± 0.5	19.5 ± 0.4	12.2 ± 1.7	49.8 ± 1.6	17.9 ± 0.6	10.0 ± 1.3	3.4 ± 0.8
**0.05**	11.2 ± 0.3	19.1 ± 0.3	14.3 ± 0.5	49.7 ± 0.9	17.7 ± 0.3	10.4 ± 0.7	3.7 ± 0.2
**0.1**	15.3 ± 0.2	18.6 ± 0.6	16.5 ± 0.8	55.1 ± 1.4	17.5 ± 0.6	11.6 ± 0.9	4.2 ± 0.3
**0.2**	19.0 ± 0.4	18.2 ± 0.3	17.6 ± 0.6	53.6 ± 1.2	17.2 ± 0.2	11.8 ± 0.5	3.7 ± 0.1
**0.5**	26.6 ± 0.2	16.8 ± 0.7	24.1 ± 0.6	55.7 ± 0.7	16.2 ± 0.7	12.6 ± 0.8	3.6 ± 0.5
**1.0**	37.2 ± 1.8	15.1 ± 0.4	31.9 ± 1.6	54.6 ± 0.6	14.6 ± 0.4	15.3 ± 0.8	3.8 ± 0.3
